# Associations of diabetes status and glucose measures with outcomes after endovascular therapy in patients with acute ischemic stroke: an analysis of the nationwide TREAT-AIS registry

**DOI:** 10.3389/fneur.2024.1351150

**Published:** 2024-05-15

**Authors:** Meng-Tsang Hsieh, Cheng-Yang Hsieh, Tzu-Hsien Yang, Sheng-Feng Sung, Yi-Chen Hsieh, Chung-Wei Lee, Chun-Jen Lin, Yu-Wei Chen, Kuan-Hung Lin, Pi-Shan Sung, Chih-Wei Tang, Hai-Jui Chu, Kun-Chang Tsai, Chao-Liang Chou, Ching-Huang Lin, Cheng-Yu Wei, Te-Yuan Chen, Shang-Yih Yan, Po-Lin Chen, Chen-Yu Hsiao, Lung Chan, Yen-Chu Huang, Hon-Man Liu, Sung-Chun Tang, I-Hui Lee, Li-Ming Lien, Hung-Yi Chiou, Jiunn-Tay Lee, Jiann-Shing Jeng

**Affiliations:** ^1^Stroke Center and Department of Neurology, Chi-Mei Medical Center, Tainan, Taiwan; ^2^Stroke Center and Department of Neurology, E-Da Hospital, Kaohsiung, Taiwan; ^3^Department of Neurology, Tainan Sin Lau Hospital, Tainan, Taiwan; ^4^Department of Radiology, Ditmanson Medical Foundation Chia-Yi Christian Hospital, Chiayi City, Taiwan; ^5^Division of Neurology, Department of Internal Medicine, Ditmanson Medical Foundation Chia-Yi Christian Hospital, Chiayi City, Taiwan; ^6^Program in Medical Neuroscience, Taipei Medical University, Taipei, Taiwan; ^7^Department of Medical Imaging, National Taiwan University Hospital, Taipei, Taiwan; ^8^Department of Neurology, Neurological Institute, Taipei Veterans General Hospital, Taipei, Taiwan; ^9^Department of Neurology, Landseed International Hospital, Taoyuan, Taiwan; ^10^Department of Neurology, Chi Mei Medical Center, Tainan, Taiwan; ^11^Department of Neurology, National Cheng Kung University Hospital, Tainan, Taiwan; ^12^Department of Neurology, Far Eastern Memorial Hospital, New Taipei City, Taiwan; ^13^Department of Neurology, En Chu Kong Hospital, New Taipei City, Taiwan; ^14^Department of Neurology, National Taiwan University Hospital, Hsinchu, Taiwan; ^15^Department of Neurology, Mackay Memorial Hospital, Taipei, Taiwan; ^16^Department of Neurology, Kaohsiung Veterans General Hospital, Kaohsiung, Taiwan; ^17^Department of Neurology, Chang Bing Show Chwan Memorial Hospital, Changhwa County, Taiwan; ^18^Department of Neurosurgery, E-Da Hospital, I-Shou University, Kaohsiung, Taiwan; ^19^Department of Neurology, Tri Service General Hospital, National Defense Medical Center, Taipei, Taiwan; ^20^Department of Neurology, Taichung Veterans General Hospital, Taichung, Taiwan; ^21^Department of Diagnostic Radiology, Shin Kong WHS Memorial Hospital, Taipei, Taiwan; ^22^Department of Neurology, Taipei Medical University–Shuang Ho Hospital, New Taipei City, Taiwan; ^23^Department of Neurology, Chang Gung University College of Medicine, Chang Gung Memorial Hospital, Chiayi, Taiwan; ^24^Department of Medical Imaging, Fu Jen Catholic University Hospital, New Taipei City, Taiwan; ^25^Department of Neurology, National Taiwan University Hospital, Taipei, Taiwan; ^26^Department of Neurology, Shin Kong WHS Memorial Hospital, Taipei, Taiwan; ^27^School of Public Health, College of Public Health, Taipei Medical University, Taipei, Taiwan; ^28^Institute of Population Health Sciences, National Health Research Institutes, Miaoli County, Taiwan

**Keywords:** acute ischemic stroke, diabetes, endovascular therapy, plasma glucose, outcomes

## Abstract

**Background:**

Hyperglycemia affects the outcomes of endovascular therapy (EVT) for acute ischemic stroke (AIS). This study compares the predictive ability of diabetes status and glucose measures on EVT outcomes using nationwide registry data.

**Methods:**

The study included 1,097 AIS patients who underwent EVT from the Taiwan Registry of Endovascular Thrombectomy for Acute Ischemic Stroke. The variables analyzed included diabetes status, admission glucose, glycated hemoglobin (HbA1c), admission glucose-to-HbA1c ratio (GAR), and outcomes such as 90-day poor functional outcome (modified Rankin Scale score ≥ 2) and symptomatic intracranial hemorrhage (SICH). Multivariable analyses investigated the independent effects of diabetes status and glucose measures on outcomes. A receiver operating characteristic (ROC) analysis was performed to compare their predictive abilities.

**Results:**

The multivariable analysis showed that individuals with known diabetes had a higher likelihood of poor functional outcomes (odds ratios [ORs] 2.10 to 2.58) and SICH (ORs 3.28 to 4.30) compared to those without diabetes. Higher quartiles of admission glucose and GAR were associated with poor functional outcomes and SICH. Higher quartiles of HbA1c were significantly associated with poor functional outcomes. However, patients in the second HbA1c quartile (5.6–5.8%) showed a non-significant tendency toward good functional outcomes compared to those in the lowest quartile (<5.6%). The ROC analysis indicated that diabetes status and admission glucose had higher predictive abilities for poor functional outcomes, while admission glucose and GAR were better predictors for SICH.

**Conclusion:**

In AIS patients undergoing EVT, diabetes status, admission glucose, and GAR were associated with 90-day poor functional outcomes and SICH. Admission glucose was likely the most suitable glucose measure for predicting outcomes after EVT.

## 1 Introduction

Endovascular therapy (EVT) is an effective treatment for acute ischemic stroke (AIS) due to large vessel occlusion, with the number needed to treat ranging from three to seven (1). However, real-world data have shown that less than half of stroke patients can achieve functional independence after undergoing EVT (2, 3). Therefore, it is crucial to find a predictor of EVT success to aid in decision making, inform prognosis, and develop new treatment strategies to improve outcomes for patients with ischemic stroke.

Hyperglycemia, whether with or without diabetes, is a well-known risk factor for stroke (4) and a potentially modifiable predictor of adverse outcomes after stroke (5–7). Pre-stroke glycemic control, as represented by glycated hemoglobin (HbA1c), and stress hyperglycemia, as defined by the plasma glucose-to-HbA1c ratio (GAR), have been shown to predict stroke outcomes after EVT (8–16). Furthermore, approximately 30% of patients with AIS have prediabetes (17), while approximately one-fifth of diabetic stroke patients are newly diagnosed with diabetes after stroke (18). Although prediabetes and newly diagnosed diabetes may predict poor outcomes in AIS patients treated with or without intravenous thrombolysis (IVT) (19–21), it remains unclear whether prediabetes or newly diagnosed diabetes after stroke are associated with adverse stroke outcomes after EVT.

Despite the abundant literature available, few studies have directly compared the effectiveness of different glucose measures and diabetes status in predicting stroke outcomes after EVT. Therefore, using a nationwide registry database, we aimed to evaluate and compare the predictive ability of diabetes status, admission glucose, HbA1c, and GAR for outcome events after EVT in patients with AIS due to large vessel occlusion.

## 2 Methods

### 2.1 Data source

This study retrospectively analyzed the data obtained from the Taiwan Registry of Endovascular Thrombectomy for Acute Ischemic Stroke (TREAT-AIS). TREAT-AIS is an ongoing nationwide multicenter registry program that prospectively enrolls adult patients with AIS who underwent EVT for large vessel occlusion in 19 hospitals across Taiwan (2). The criteria for EVT follow the guidelines published by the American Heart Association/American Stroke Association (22) and the Taiwan Stroke Society (23). The registry received approval from the Joint Institutional Review Board of Taipei Medical University and the Institutional Review Boards of all participating hospitals.

The TREAT-AIS program prospectively collects information on demographics, risk factors, and stroke etiology based on the Trial of ORG 10172 in Acute Stroke Treatment classification, laboratory tests, imaging studies, medications, surgical interventions, complications, National Institutes of Health Stroke Scale (NIHSS) scores at arrival, before needle insertion, and 24 h after EVT, and modified Rankin Scale (mRS) scores at discharge and 90 days post-stroke.

The following time points for EVT procedures are recorded: last known well, arrival at the emergency department, initial imaging study, IVT if applicable, arterial puncture, and reperfusion. Additionally, devices used (such as stent retrievers, thrombosuction, and others), the number of passes, and the extent of recanalization are recorded. Reperfusion success is defined as a modified thrombolysis in a cerebral infarction score of 2b or 3.

### 2.2 Study population

The study population included patients who met the following inclusion criteria: (i) aged ≥20 years; (ii) with confirmed large vessel occlusion by computed tomography or magnetic resonance angiography; and (iii) undergoing EVT between January 2019 and June 2022. Patients with missing glucose status or outcome events were excluded from the study.

### 2.3 Diabetes status and glucose measures

Patients were grouped into those with known diabetes, newly diagnosed diabetes, prediabetes, and non-diabetes. The known diabetes group included patients with a pre-stroke existing diagnosis of diabetes, with or without the use of antidiabetic medication. The newly diagnosed diabetes group included patients without pre-existing diabetes but with an HbA1c level of ≥6.5%. Patients with an HbA1c level between 5.7 and 6.4% were assigned to the pre-diabetes group, while the remaining patients were assigned to the non-diabetes group.

Three different glucose measures were investigated in this study. The first glucose measure was the plasma glucose level upon arrival after stroke onset. The second measure was the first HbA1c level obtained within 48 h after the stroke. Finally, the GAR (24) or stress hyperglycemia ratio (16), calculated as the ratio of plasma glucose to HbA1c, was used as a third glucose measure. For these glucose measures, patients were categorized according to quartiles of the levels of plasma glucose, HbA1c, and GAR, respectively.

### 2.4 Outcome events

The primary outcome event was the functional outcome at 90 days as assessed using the mRS. The mRS score was dichotomized into 0–2 (good functional outcome) versus 3–6 (poor functional outcome). Secondary outcome events included symptomatic intracranial hemorrhage (SICH) and reperfusion success. SICH was defined as the occurrence of new intracranial hemorrhage within 36 h of stroke onset, meeting the criteria for type 2 parenchymal hemorrhage and accompanied by an increase of ≥4 points on the NIHSS (2).

### 2.5 Statistical analysis

We used descriptive statistics to assess the characteristics of the study population. Categorical variables are presented as counts and percentages, while continuous variables are presented as means and standard deviations. We compared differences between patient groups stratified by different outcome events. The chi-square tests were used for categorical variables, and the ANOVA tests were used for continuous variables.

We performed univariable and multivariable logistic regression analyses to evaluate the association between diabetes status or each glucose measure and the outcome event. Four successive models were tested, including an unadjusted model (Model 1), a model adjusted for age, sex, and NIHSS score (Model 2), a model adjusted for age, sex, NIHSS score, and reperfusion success (Model 3), and a model adjusted for age, sex, NIHSS score, reperfusion success, and last known well to reperfusion time (Model 4). For the outcome event of reperfusion success, only Models 1 and 2 were evaluated.

In the sensitivity analysis, we investigated whether the territory of arterial occlusion and the use of intravenous thrombolysis could alter the predictive value of diabetes status or glucose measures. We classified the area of arterial occlusion into two categories: anterior circulation stroke and posterior circulation stroke. We incorporated either anterior circulation stroke or intravenous thrombolysis as a covariate in Models 2 to 4. Additionally, we carried out subgroup analyses to evaluate the predictive value of glucose measures in non-diabetic patients and in male and female patients separately.

Receiver operating characteristic (ROC) analysis was used to determine the ability of diabetes status or each glucose measure to predict the outcome event. Plasma glucose, HbA1c, and GAR were analyzed as categorical or continuous variables. We calculated the area under the receiver operating characteristic curve (AUC) and compared them using DeLong’s method (25).

We used SAS software version 9.4 (SAS Institute, Cary, NC) for all statistical analyses. A two-tailed *p*-value of 0.05 was considered significant.

## 3 Results

### 3.1 Characteristics of the study population

A total of 1,522 patients met the inclusion criteria during the study period. After removing patients with missing glucose status (*n* = 235) and those who did not have data on outcome events (*n* = 190), the study population consisted of 1,097 patients. Among them, 338 individuals had known diabetes before stroke, 88 were newly diagnosed with diabetes, 331 were prediabetic, and 340 were non-diabetic. Regarding the categories of glucose measures, the admission glucose values were divided into the following categories: <6.11 mmol/L, 6.11–7.20 mmol/L, 7.21–9.04 mmol/L, and ≥ 9.05 mmol/L. HbA1c levels were categorized as follows: <5.6%, 5.6–5.8%, 5.9–6.5%, and ≥ 6.6%. The GAR values were divided into <19.0, 19.0–21.9, 22.0–25.9, and ≥ 26.0.

### 3.2 Outcomes

Among the study population, 733 (66.8%) patients had poor functional outcomes at 90 days, 40 (3.6%) experienced SICH, and 372 (33.9%) had asymptomatic intracranial hemorrhage. Compared to patients with good functional outcomes (Table 1), those with poor functional outcomes were older, more likely to be female, and had a higher prevalence of hypertension, atrial fibrillation, and previous strokes but a lower proportion of smoking habits. Additionally, they presented with higher baseline systolic blood pressure and NIHSS scores, were less likely to receive IVT, experienced a longer time from last known well to groin puncture and reperfusion, and had a lower likelihood of achieving reperfusion success. Patients with poor functional outcomes were more likely to be diabetic and had higher levels of admission glucose, HbA1c, and GAR than those with good functional outcomes.

**Table 1:** Characteristics of the study population.

	mRS 0–2 *n* = 364	mRS 3–6 *n* = 733	*p*	No SICH *n* = 1,057	SICH *n* = 40	*p*
Age	66.2 (12.6)	73.9 (13.2)	<0.001	71.3 (13.6)	72.9 (10.9)	0.451
Female patients	135 (37.1)	361 (49.2)	<0.001	478 (45.2)	18 (45.0)	0.978
Risk factors						
Hypertension	249 (68.4)	556 (75.9)	0.009	771 (72.9)	34 (85.0)	0.090
AF	172 (47.3)	407 (55.5)	0.010	555 (52.5)	24 (60.0)	0.351
Hyperlipidemia	191 (52.5)	385 (52.5)	0.987	557 (52.7)	19 (47.5)	0.518
Smoking	132 (36.3)	187 (25.5)	<0.001	310 (29.3)	9 (22.5)	0.351
Prior stroke	58 (15.9)	174 (23.7)	0.003	221 (20.9)	11 (27.5)	0.316
SBP, mmHg	150.3 (28.7)	154.3 (30.3)	0.039	153 (29.9)	151.5 (27.7)	0.750
DBP, mmHg	87.1 (19.2)	86.7 (19.7)	0.709	86.7 (19.4)	89.6 (21.1)	0.352
Initial NIHSS score	14.8 (6.9)	19.1 (6.9)	<0.001	17.6 (7.2)	20.2 (6.4)	0.024
Anterior circulation stroke	318 (87.4)	618 (84.3)	0.179	903 (85.4)	33 (82.5)	0.607
TOAST			0.368			0.440
LAA	125 (34.3)	238 (32.5)		353 (33.4)	10 (25.0)	
CE	171 (47)	331 (45.2)		483 (45.7)	19 (47.5)	
Others	68 (18.7)	164 (22.4)		221 (20.9)	11 (27.5)	
Intravenous thrombolysis	169 (46.6)	248 (33.9)	<0.001	400 (37.9)	17 (42.5)	0.558
EVT procedure						
LKW to puncture time, min	292 (211)	355 (253)	<0.001	335 (241)	321 (257)	0.739
LKW to reperfusion time, min	306 (193)	369 (234)	<0.001	346 (220)	377 (274)	0.430
Use of stent retriever	96 (26.4)	224 (30.6)	0.151	307 (29.0)	13 (32.5)	0.637
Pass number	1.9 (1.2)	2.1 (1.4)	0.447	2.0 (1.4)	1.9 (1.1)	0.769
Reperfusion success	339 (93.1)	581 (79.3)	<0.001	889 (84.1)	31 (77.5)	0.265
Diabetes status			<0.001			0.020
Non-diabetic	138 (37.9)	202 (27.6)		335 (31.7)	5 (12.5)	
Prediabetic	125 (34.3)	206 (28.1)		320 (30.3)	11 (27.5)	
Newly diagnosed diabetic	25 (6.9)	63 (8.6)		84 (7.9)	4 (10.0)	
Known diabetic	76 (20.9)	262 (35.7)		318 (30.1)	20 (50.0)	
Admission glucose, mmol/L	7.56 (2.96)	8.45 (3.55)	<0.001	146.2 (61.5)	168.2 (49.3)	0.026
HbA1c, %	6.1 (1.2)	6.4 (1.4)	0.004	6.3 (1.4)	6.3 (0.8)	0.922
GAR	22.0 (5.4)	23.5 (6.3)	<0.001	22.9 (6.0)	26.5 (6.9)	<0.001

Data are given as *n* (%) and mean (standard deviation).AF, atrial fibrillation; CE, cardioembolism; DBP, diastolic blood pressure; EVT, endovascular therapy; GAR, glucose-to-glycated hemoglobin ratio; HbA1c, glycated hemoglobin; LAA, large artery atherosclerosis; LKW, last known well; NIHSS, National Institutes of Health Stroke Scale; SBP, systolic blood pressure; TOAST, Trial of ORG 10172 in Acute Stroke Treatment.

Compared to patients without SICH (Table 1), those with SICH had higher initial NIHSS scores and were more likely to be diabetic. Additionally, they had higher levels of admission glucose and GAR. However, no significant differences were observed for other risk factors or laboratory results between patients with SICH and those without.

### 3.3 Predictive ability of diabetes status and glucose measures

**Figure 1:**
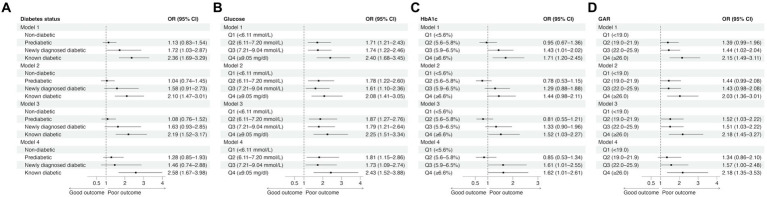
The effects of diabetes status **(A)** and glucose measures **(B–D)** on 90-day functional outcomes in univariable (Model 1) and multivariable logistic regression analyses (Model 2: adjusted for age, sex, and NIHSS score; Model 3: adjusted for age, sex, NIHSS score, and reperfusion success; Model 4: adjusted for age, sex, NIHSS score, reperfusion success, and last known well to reperfusion time). CI, confidence interval; GAR, glucose-to-HbA1c ratio; HbA1c, glycated hemoglobin; OR, odds ratio.

In the unadjusted model (Model 1), patients with known diabetes and those with newly diagnosed diabetes were significantly more likely to experience poor outcomes at 90 days than non-diabetic patients (Figure 1A; Supplementary Table S1). In the adjusted models (Models 2 to 4), only known diabetics showed a significantly higher likelihood of poor outcomes at 90 days than non-diabetics, with odds ratios (ORs) ranging from 2.10 to 2.58.

Higher quartiles of admission glucose were consistently associated with a significantly increased risk of poor functional outcomes across different models (Figure 1B; Supplementary Table S1). In contrast, while patients in the third (5.9–6.5%) and fourth (≥6.6%) HbA1c quartiles were more likely to experience poor outcomes (Figure 1C; Supplementary Table S1), the association was relatively weak. Notably, patients in the second quartile (5.6–5.8%) had a non-significant tendency toward good outcomes compared to those in the lowest HbA1c quartile (<5.6%). On the other hand, higher GAR quartiles were associated with poor outcomes. Patients in the fourth GAR quartile (≥26.0) had a consistently significantly higher risk of poor outcomes than those in the lowest GAR quartile (<19.0) (Figure 1D; Supplementary Table S1).

**Figure 2:**
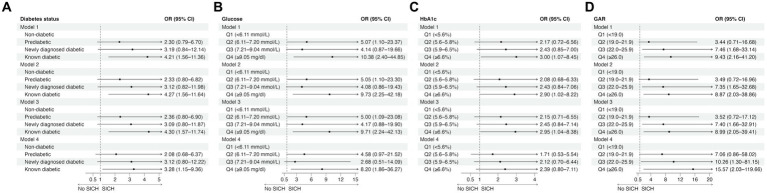
The effects of diabetes status **(A)** and glucose measures **(B–D)** on symptomatic intracranial hemorrhage in univariable (Model 1) and multivariable logistic regression analyses (Model 2: adjusted for age, sex, and NIHSS score; Model 3: adjusted for age, sex, NIHSS score, and reperfusion success; Model 4: adjusted for age, sex, NIHSS score, reperfusion success, and last known well to reperfusion time). CI, confidence interval; GAR, glucose-to-HbA1c ratio; HbA1c, glycated hemoglobin; OR, odds ratio; SICH, symptomatic intracranial hemorrhage.

Regarding SICH, patients with known diabetes had a significantly higher risk of SICH than non-diabetics in all four models (Figure 2A; Supplementary Table S2). Patients in the fourth glucose quartile (≥9.05 mmol/L) had a significantly higher risk of SICH than those in the first glucose quartile (<6.11 mmol/L) (Figure 2B; Supplementary Table S2). Patients in the fourth HbA1c quartile (≥6.6%) had a significantly higher likelihood of SICH compared to those in the lowest HbA1c quartile (<5.6%) in Models 1, 2, and 3 but not in Model 4 (Figure 2C; Supplementary Table S2). However, there was a trend toward higher odds of SICH for higher HbA1c levels. Similarly, a trend toward higher odds of SICH was observed for higher GAR. Additionally, patients in the third (22.0–25.9) and fourth (≥26.0) GAR quartiles had a significantly higher risk of SICH than those in the lowest GAR quartile (<19.0) in all four models (Figure 2D; Supplementary Table S2).

As for reperfusion success (Supplementary Table S3), none of the diabetes status and glucose measures were associated with reperfusion success in either the unadjusted model or the model adjusted for age, sex, and initial NIHSS.

Table 2 and Supplementary Table S4 display the AUC values for diabetes status and various glucose measures. When glucose measures were analyzed as categorical variables, diabetes status had the highest AUC for predicting poor outcomes, followed by admission glucose. However, the difference was not statistically significant (*p* = 0.602 for Model 1; *p* = 0.565 for Model 2; *p* = 0.601 for Model 3; *p* = 0.708 for Model 4). For the prediction of SICH, admission glucose exhibited the highest AUC in Model 1, while GAR achieved the highest AUC in Models 2 to 4. HbA1c had the lowest AUC in all models. The AUC of HbA1c was significantly lower than that of GAR (*p* = 0.014 for Model 4) and that of admission glucose (*p* = 0.042 for Model 1; *p* = 0.042 for Model 3; *p* = 0.016 for Model 4).

**Table 2:** AUC values for diabetes status and different glucose measures.

	Categorical	Continuous
Poor outcomes		
Diabetes status	0.592 (0.559–0.626)	NA
Admission glucose	0.583 (0.548–0.618)	0.593 (0.558–0.629)
HbA1c	0.565 (0.530–0.600)	0.567 (0.531–0.602)
GAR	0.571 (0.536–0.605)	0.574 (0.538–0.609)
SICH		
Diabetes status	0.639 (0.561–0.717)	NA
Admission glucose	0.678 (0.603–0.752)	0.673 (0.597–0.749)
HbA1c	0.594 (0.512–0.676)	0.583 (0.505–0.662)
GAR	0.676 (0.605–0.747)	0.674 (0.594–0.753)

AUC, area under the receiver operating characteristic curve; GAR, glucose-to-glycated hemoglobin ratio; HbA1c, glycated hemoglobin.

When glucose measures were analyzed as continuous variables, admission glucose achieved the highest AUC for predicting poor outcomes, followed by GAR and HbA1c. The AUC of admission glucose was significantly higher than that of GAR (*p* = 0.032). GAR had the highest AUC for predicting SICH, followed closely by admission glucose, while HbA1c had the lowest AUC. However, the difference in AUCs between GAR and admission glucose was not significant (*p* = 0.987).

### 3.4 Sensitivity and subgroup analyses

In the sensitivity analysis, adding anterior circulation stroke or intravenous thrombolysis to the models did not materially alter the results. Specifically, known diabetes, higher admission glucose quartiles, the third and fourth HbA1c quartiles, and GAR were associated with poor outcomes (Supplementary Figures S1, S3). Known diabetes, the fourth quartile of admission glucose and HbA1c, and the third and fourth GAR quartiles were linked to SICH (Supplementary Figures S2, S4).

In the non-diabetic subgroup, the fourth quartile of admission glucose was linked to poor functional outcomes. However, no significant correlation was found between admission glucose and SICH (Supplementary Figure S5).

In male patients, known diabetes and the fourth quartile of admission glucose and GAR were associated with poor functional outcomes. Higher HbA1c quartiles showed a trend toward poor outcomes, but this was not statistically significant (Supplementary Figure S6). The fourth quartile of admission glucose and the third and fourth quartiles of GAR were associated with a higher likelihood of SICH (Supplementary Figure S7).

In female patients, known diabetes and higher admission glucose quartiles were linked to poor functional outcomes. Much like their male counterparts, higher HbA1c quartiles showed a trend toward poor outcomes, but it was not statistically significant (Supplementary Figure S8). A detailed analysis of SICH in this subgroup was not possible due to the small number of events (Supplementary Figure S9). In summary, we did not identify a sex difference in the correlation between glucose measures and stroke outcomes.

## 4 Discussion

We found that diabetes status, admission glucose, and GAR were associated with poor functional outcomes and SICH in patients with AIS undergoing EVT. By contrast, the association between HbA1C and poor functional outcomes, or SICH, was borderline. Interestingly, HbA1c appeared to have a non-linear relationship with the risk of poor functional outcomes. The ROC analysis indicated that diabetes status and admission glucose had similar abilities to predict poor functional outcomes, while admission glucose and GAR had similar predictive abilities for SICH. HbA1c had the lowest predictive ability for poor functional outcomes and SICH.

### 4.1 Diabetes status and outcomes

In accordance with previous studies (6, 26), we discovered that known diabetes was linked to a higher risk of functional dependence following EVT. Conversely, while the connection between known diabetes and SICH varied in the literature (6, 26, 27), this study showed an increased risk of post-treatment SICH in individuals with known diabetes, similar to those treated with IVT (28). By contrast, newly diagnosed diabetes, defined by a single high HbA1c, was not associated with adverse functional outcomes or SICH in this study. A Chinese study on unselected patients with AIS also found no association between newly diagnosed diabetes (defined by the same criteria) and poor functional outcomes (29). This suggests that the current HbA1c threshold for diagnosing diabetes may not accurately predict outcomes in Asian AIS patients (29). However, a Korean study primarily focusing on AIS patients at high risk of cerebral hemorrhage indicated that newly diagnosed diabetes significantly increased the risk of post-stroke cardiovascular events (21). Additionally, prediabetes did not correlate with poor functional outcomes or SICH in our EVT patients. Previous studies have similarly shown no association between prediabetes and poor functional outcomes at various time points after stroke in unselected AIS patients (20) or those treated with IVT (19).

### 4.2 Admission glucose, HbA1c, GAR, and outcomes

Consistent with the literature (6, 7), admission glucose was significantly associated with poor functional outcomes and SICH after EVT in our patients. Admission hyperglycemia may modify the effect of EVT on stroke outcomes, with the benefit of EVT decreasing as glucose levels increase (30). The detrimental effects of hyperglycemia may be mediated by direct tissue injury caused by mitochondrial dysfunction and lactic acidosis, impaired recanalization, decreased reperfusion, and increased reperfusion injury (31). In addition, oxidative and nitrosative stress mechanisms, mediated by peroxynitrite, may also have a significant impact on the worsening of stroke due to hyperglycemia, as suggested by preclinical studies (32, 33).

By contrast, HbA1c, which measures baseline glycemic control over the past 3 months (34), had the lowest AUCs in predicting poor functional outcomes and SICH. Additionally, compared to patients in the lowest HbA1c quartile (<5.6%), those in the second HbA1c quartile (5.6–5.8%) showed a trend toward good outcomes (Figure 1C), suggesting a possible non-linear relationship between HbA1c and functional outcomes. A Korean study also found that patients in the lowest and highest HbA1c groups had a higher risk of poor functional outcomes after EVT than those in the middle groups (10). Similar J-shaped or U-shaped relationships have been observed in patients with acute myocardial infarction undergoing percutaneous coronary intervention, where both low HbA1c and high HbA1c were associated with an increased risk of major adverse cardiovascular events and mortality (35, 36). These observations may be attributed to the negative effect of hypoglycemia in patients with low HbA1c due to excessive glycemic control. The non-linear relationship between HbA1c and stroke outcomes may explain why HbA1c achieved the lowest AUC.

On the other hand, GAR achieved comparable AUC values to admission glucose, especially in the prediction of SICH. These findings are consistent with previous studies that examined unselected AIS patients with or without thrombolysis (37, 38). The reason for using GAR to account for HbA1c is that chronic hyperglycemia could downregulate glucose transporters (39). This would lead to reduced sensitivity of the neuroendocrine system to stress and minimize the damage caused by stress hyperglycemia during acute stroke. However, we did not observe any additional benefit when using GAR instead of admission glucose in predicting outcome after EVT. It is worth noting that GAR was calculated in this study using random admission glucose rather than fasting glucose, as in other studies (11–15).

### 4.3 Clinical implications

In addition to diabetes status, this nationwide registry study suggests that admission glucose may be the most suitable glucose measure for predicting functional outcomes and SICH after EVT. Unlike diabetes history, plasma glucose measurement is readily available, even in comatose patients, and it is a recommended routine laboratory test for the management of acute stroke (22).

Admission glucose can be considered a simple mixed indicator of background glycemia and hyperglycemic reaction to stress during acute stroke. In contrast, obtaining HbA1c and GAR in the acute setting may require additional cost and effort, and these two glucose measures did not provide better outcome prediction than admission glucose. Although GAR calculated using fasting plasma glucose may perform better than using random admission glucose in predicting functional outcomes after IVT (37), it is unlikely to obtain fasting glucose and make informed decisions based on the prediction within the short time window before EVT. Moreover, fasting plasma glucose may be affected by post-stroke management, such as glycemic control in the stroke unit.

Currently, EVT has become the standard treatment for AIS patients with large vessel occlusion (22). However, more real-world studies are still needed to better understand the effectiveness and safety of EVT outside the controlled clinical trial settings (3). Such studies are particularly important for identifying subgroups of patients who may benefit the most from EVT. Since diabetes status or glucose level significantly predicts stroke outcomes, proper adjustment for this factor is crucial for these real-world studies. Our study findings may provide insights for future studies on which glucose measure to use and how to incorporate it for multivariable adjustment. For example, considering the non-linear relationship between HbA1c and functional outcomes, treating HbA1C as a categorical covariate may be more appropriate. Additionally, current risk scores for predicting outcomes of EVT typically include admission glucose as one of the elements (40, 41). Diabetes status, or GAR, can be considered an alternative predictor when developing or refining these risk scores.

### 4.4 Limitations

This study has several limitations worth mentioning. First, there were only 88 patients with newly diagnosed diabetes, so we cannot rule out the possibility that the non-significant association between newly diagnosed diabetes and outcomes was due to insufficient statistical power. Second, we did not control for the type of antidiabetic drugs used before stroke in the multivariable analysis. Pre-stroke exposure to sulfonylureas may decrease the likelihood of favorable functional outcomes, especially in non-lacunar stroke subtypes (42). However, this issue remains debated, as evidenced by several studies showing that administering sulfonylurea before a stroke has no impact on the severity or outcomes of the stroke (43–45). Third, the percentage of poor functional outcomes in TREAT-AIS was higher than in other registries, likely due to a longer door-to-puncture time (2). The possibility that a high percentage of poor functional outcomes may influence the predictive ability of glucose measures and limit the generalizability of the study cannot be ruled out.

## 5 Conclusion

This study examined the association between diabetes status, admission glucose, HbA1c, and GAR with functional outcomes and SICH in patients with AIS undergoing EVT. The results indicated that diabetes status, admission glucose, and GAR were associated with poor functional outcomes and SICH. The association between HbA1c and outcomes was only marginally significant. Admission glucose was found to be the most suitable glucose measure for predicting outcomes after EVT. However, diabetes status and GAR can be considered as alternative measures for outcome prediction. This study also suggests a potential non-linear relationship between HbA1c and functional outcomes. These findings have clinical implications for the management of AIS patients undergoing EVT. In addition, future studies investigating the effectiveness of EVT using real-world data should take into account the varying predictive abilities of diabetes status and different glucose measures.

## Data availability statement

The data analyzed in this study is subject to the following licenses/restrictions: Data privacy regulation rule of our country. Requests to access these datasets should be directed to Sung-Chun Tang, tangneuro@gmail.com.

## Author contributions

M-TH: Writing – review & editing, Writing – original draft, Investigation, Formal analysis, Data curation, Conceptualization. C-YaH: Writing – review & editing, Writing – original draft, Validation, Methodology, Investigation, Conceptualization. T-HY: Writing – review & editing, Project administration, Formal analysis, Data curation. S-FS: Writing – review & editing, Writing – original draft, Visualization, Validation, Project administration, Methodology, Investigation, Formal analysis. Y-CheH: Writing – review & editing, Supervision, Formal analysis, Data curation. C-WL: Writing – review & editing, Data curation. C-JL: Writing – review & editing, Data curation. Y-WC: Writing – review & editing, Data curation. K-HL: Writing – review & editing, Data curation. P-SS: Writing – review & editing, Data curation. C-WT: Writing – review & editing, Data curation. H-JC: Writing – review & editing, Data curation. K-CT: Writing – review & editing, Data curation. C-LC: Writing – review & editing, Data curation. C-HL: Writing – review & editing, Data curation. C-YW: Writing – review & editing, Data curation. T-YC: Writing – review & editing, Data curation. S-YY: Writing – review & editing, Data curation. P-LC: Writing – review & editing, Data curation. C-YuH: Writing – review & editing, Data curation. LC: Writing – review & editing, Data curation. Y-ChuH: Writing – review & editing, Data curation. H-ML: Writing – review & editing, Data curation. S-CT: Writing – review & editing, Validation, Supervision, Resources, Investigation, Funding acquisition, Formal analysis, Data curation. I-HL: Writing – review & editing, Data curation. L-ML: Writing – review & editing, Supervision, Resources. H-YC: Writing – review & editing, Supervision. J-TL: Writing – review & editing, Supervision. J-SJ: Writing – review & editing, Supervision.

## Ethics statement

The studies involving humans were approved by the registry received approval from the Joint Institutional Review Board of Taipei Medical University and the Institutional Review Board of all participating hospitals. The studies were conducted in accordance with the local legislation and institutional requirements. Written informed consent for participation was not required from the participants or the participants' legal guardians/next of kin in accordance with the national legislation and institutional requirements.
