# Supplementary material for: Associations of diabetes status and glucose measures with outcomes after endovascular therapy in patients with acute ischemic stroke: an analysis of the nationwide TREAT-AIS registry
Source: Front Neurol. 2024 May 15;15:1351150. doi: 10.3389/fneur.2024.1351150 (PMC11135283; doi:10.3389/fneur.2024.1351150)
Supplement: Supplementary file 1 [file Data_Sheet_1.pdf]

Supplementary Tables

Supplementary Table 1. Risk of poor functional outcomes at 90 days stratified by diabetes status and each glucose measure.

	N (%)	Model 1		Model 2		Model 3		Model 4	
		OR (95% CI)	*P*	OR (95% CI)	*P*	OR (95% CI)	*P*	OR (95% CI)	*P*
Diabetes status									
Non-diabetic	340 (31.0)	Ref		Ref		Ref		Ref	
Prediabetic	331 (30.2)	1.13 (0.83–1.54)	0.454	1.04 (0.74–1.45)	0.840	1.08 (0.76–1.52)	0.681	1.28 (0.85–1.93)	0.247
Newly diagnosed diabetic	88 (8.0)	1.72 (1.03–2.87)	0.037	1.58 (0.91–2.73)	0.102	1.63 (0.93–2.85)	0.085	1.46 (0.74–2.88)	0.276
Known diabetic	338 (30.8)	2.36 (1.69–3.29)	<0.001	2.10 (1.47–3.01)	<0.001	2.19 (1.52–3.17)	<0.001	2.58 (1.67–3.98)	<0.001
Admission glucose									
Q1 (<6.11 mmol/L)	272 (24.79)	Ref		Ref		Ref		Ref	
Q2 (6.11–7.20 mmol/L)	276 (25.16)	1.71 (1.21–2.43)	0.003	1.78 (1.22–2.60)	0.003	1.87 (1.27–2.76)	0.002	1.81 (1.15–2.86)	0.010
Q3 (7.21–9.04 mmol/L)	269 (24.52)	1.74 (1.22–2.46)	0.002	1.61 (1.10–2.36)	0.014	1.79 (1.21–2.64)	0.003	1.73 (1.09–2.74)	0.019
Q4 (≥9.05 mmol/L)	280 (25.52)	2.40 (1.68–3.45)	<0.001	2.08 (1.41–3.05)	<0.001	2.25 (1.51–3.34)	<0.001	2.43 (1.52–3.88)	<0.001
HbA1c									
Q1 (<5.6%)	287 (26.16)	Ref		Ref		Ref		Ref	
Q2 (5.6–5.8%)	243 (22.15)	0.95 (0.671.36)	0.793	0.78 (0.53–1.15)	0.208	0.81 (0.55–1.21)	0.302	0.85 (0.53–1.34)	0.473
Q3 (5.9–6.5%)	290 (26.44)	1.43 (1.01–2.02)	0.043	1.29 (0.88–1.88)	0.190	1.33 (0.90–1.96)	0.155	1.61 (1.01–2.55)	0.043
Q4 (≥6.6%)	277 (25.25)	1.71 (1.20–2.45)	0.003	1.44 (0.98–2.11)	0.065	1.52 (1.03–2.27)	0.037	1.62 (1.01–2.61)	0.045
GAR									
Q1 (<19.0)	276 (25.16)	Ref		Ref		Ref		Ref	
Q2 (19.0–21.9)	286 (26.07)	1.39 (0.99–1.96)	0.058	1.44 (0.99–2.08)	0.057	1.52 (1.03–2.22)	0.034	1.34 (0.86–2.10)	0.202
Q3 (22.0–25.9)	271 (24.70)	1.44 (1.02–2.04)	0.041	1.43 (0.98–2.08)	0.064	1.51 (1.03–2.22)	0.036	1.57 (1.00–2.48)	0.052
Q4 (≥26.0)	264 (24.07)	2.15 (1.49–3.11)	<0.001	2.03 (1.36–3.01)	<0.001	2.18 (1.45–3.27)	<0.001	2.18 (1.35–3.53)	0.002

CI, confidence interval; GAR, glucose-to-glycated hemoglobin ratio; HbA1c, glycated hemoglobin; OR, odds ratio.

Supplementary Table 2. Risk of symptomatic intracranial hemorrhage stratified by diabetes status and each glucose measure.

	N (%)	Model 1		Model 2		Model 3		Model 4	
		OR (95% CI)	*P*	OR (95% CI)	*P*	OR (95% CI)	*P*	OR (95% CI)	*P*
Diabetes status									
Non-diabetic	340 (31.0)	Ref		Ref		Ref		Ref	
Prediabetic	331 (30.2)	2.30 (0.79–6.70)	0.126	2.33 (0.80–6.82)	0.123	2.36 (0.80–6.90)	0.118	2.08 (0.68–6.37)	0.198
Newly diagnosed diabetic	88 (8.0)	3.19 (0.84–12.14)	0.089	3.12 (0.82–11.98)	0.097	3.09 (0.80–11.87)	0.101	3.12 (0.80–12.22)	0.102
Known diabetic	338 (30.8)	4.21 (1.56–11.36)	0.005	4.27 (1.56–11.64)	0.005	4.30 (1.57–11.74)	0.004	3.28 (1.15–9.36)	0.026
Admission glucose									
Q1 (<6.11 mmol/L)	272 (24.79)	Ref		Ref		Ref		Ref	
Q2 (6.11–7.20 mmol/L)	276 (25.16)	5.07 (1.10–23.37)	0.037	5.05 (1.10–23.30)	0.038	5.00 (1.09–23.08)	0.039	4.58 (0.97–21.52)	0.054
Q3 (7.21–9.04 mmol/L)	269 (24.52)	4.14 (0.87–19.66)	0.074	4.08 (0.86–19.43)	0.078	4.17 (0.88–19.90)	0.073	2.68 (0.51–14.09)	0.243
Q4 (≥9.05 mmol/L)	280 (25.52)	10.38 (2.40–44.85)	0.002	9.73 (2.25–42.18)	0.002	9.71 (2.24–42.13)	0.002	8.20 (1.86–36.27)	0.006
HbA1c									
Q1 (<5.6%)	287 (26.16)	Ref		Ref		Ref		Ref	
Q2 (5.6–5.8%)	243 (22.15)	2.17 (0.72–6.56)	0.170	2.08 (0.68–6.33)	0.197	2.15 (0.71–6.55)	0.178	1.71 (0.53–5.54)	0.369
Q3 (5.9–6.5%)	290 (26.44)	2.43 (0.85–7.00)	0.099	2.43 (0.84–7.06)	0.103	2.45 (0.84–7.14)	0.100	2.12 (0.70–6.44)	0.183
Q4 (≥6.6%)	277 (25.25)	3.00 (1.07–8.45)	0.037	2.90 (1.02–8.22)	0.045	2.95 (1.04–8.38)	0.042	2.39 (0.80–7.11)	0.118
GAR									
Q1 (<19.0)	276 (25.16)	Ref		Ref		Ref		Ref	
Q2 (19.0–21.9)	286 (26.07)	3.44 (0.71–16.68)	0.126	3.49 (0.72–16.96)	0.122	3.52 (0.72–17.12)	0.119	7.06 (0.86–58.02)	0.069
Q3 (22.0–25.9)	271 (24.70)	7.46 (1.68–33.14)	0.008	7.35 (1.65–32.68)	0.009	7.40 (1.66–32.91)	0.009	10.26 (1.30–81.15)	0.027
Q4 (≥26.0)	264 (24.07)	9.43 (2.16–41.20)	0.003	8.87 (2.03–38.86)	0.004	8.99 (2.05–39.41)	0.004	15.57 (2.03–119.66)	0.008

CI, confidence interval; GAR, glucose-to-glycated hemoglobin ratio; HbA1c, glycated hemoglobin; OR, odds ratio.

Supplementary Table 3. Successful reperfusion stratified by diabetes status and each glucose measure.

	N (%)	Model 1		Model 2	
		OR (95% CI)	*P*	OR (95% CI)	*P*
Diabetes status					
Non-diabetic	340 (31.0)	Ref		Ref	
Prediabetic	331 (30.2)	1.18 (0.78-1.79)	0.425	1.16 (0.77-1.77)	0.481
Newly diagnosed diabetic	88 (8.0)	1.00 (0.54-1.87)	0.998	0.97 (0.52-1.82)	0.924
Known diabetic	338 (30.8)	1.06 (0.71-1.59)	0.784	1.05 (0.69-1.58)	0.833
Admission glucose					
Q1 (<6.11 mmol/L)	272 (24.79)	Ref		Ref	
Q2 (6.11–7.20 mmol/L)	276 (25.16)	0.99 (0.64-1.54)	0.974	0.97 (0.62-1.50)	0.875
Q3 (7.21–9.04 mmol/L)	269 (24.52)	1.48 (0.92-2.38)	0.106	1.47 (0.91-2.38)	0.112
Q4 (≥9.05 mmol/L)	280 (25.52)	1.09 (0.70-1.70)	0.703	1.07 (0.68-1.67)	0.779
HbA1c					
Q1 (<5.6%)	287 (26.16)	Ref		Ref	
Q2 (5.6–5.8%)	243 (22.15)	1.53 (0.95-2.46)	0.080	1.49 (0.93-2.41)	0.100
Q3 (5.9–6.5%)	290 (26.44)	1.14 (0.74-1.75)	0.547	1.12 (0.72-1.72)	0.624
Q4 (≥6.6%)	277 (25.25)	1.30 (0.83-2.02)	0.249	1.27 (0.82-1.99)	0.288
GAR					
Q1 (<19.0)	276 (25.16)	Ref		Ref	
Q2 (19.0–21.9)	286 (26.07)	1.16 (0.74-1.80)	0.522	1.14 (0.73-1.79)	0.552
Q3 (22.0–25.9)	271 (24.70)	1.15 (0.73-1.79)	0.556	1.15 (0.73-1.80)	0.553
Q4 (≥26.0)	264 (24.07)	1.21 (0.77-1.91)	0.416	1.19 (0.75-1.88)	0.457

CI, confidence interval; GAR, glucose-to-glycated hemoglobin ratio; HbA1c, glycated hemoglobin; OR, odds ratio.

Supplementary Table 4. AUC values for diabetes status and different glucose measures.

	Model 1	Model 2	Model 3	Model 4
Poor outcomes				
Diabetes status	0.592 (0.559-0.626)	0.739 (0.709-0.769)	0.768 (0.740-0.796)	0.789 (0.757-0.821)
Admission glucose	0.583 (0.548-0.618)	0.735 (0.705-0.765)	0.765 (0.737-0.794)	0.787 (0.755-0.819)
HbA1c	0.565 (0.530-0.600)	0.733 (0.702-0.763)	0.762 (0.734-0.791)	0.784 (0.752-0.816)
GAR	0.571 (0.536-0.605)	0.731 (0.701-0.762)	0.764 (0.735-0.792)	0.784 (0.752-0.816)
SICH				
Diabetes status	0.639 (0.561-0.717)	0.690 (0.605-0.775)	0.693 (0.610-0.777)	0.680 (0.587-0.774)
Admission glucose	0.678 (0.603-0.752)	0.712 (0.632-0.793)	0.717 (0.636-0.797)	0.731 (0.643-0.818)
HbA1c	0.594 (0.512-0.676)	0.658 (0.570-0.746)	0.655 (0.569-0.741)	0.646 (0.555-0.738)
GAR	0.676 (0.605-0.747)	0.714 (0.638-0.790)	0.722 (0.646-0.797)	0.737 (0.656-0.818)

AUC, area under the receiver operating characteristic curve; GAR, glucose-to-glycated hemoglobin ratio; HbA1c, glycated hemoglobin.

Supplementary Figures

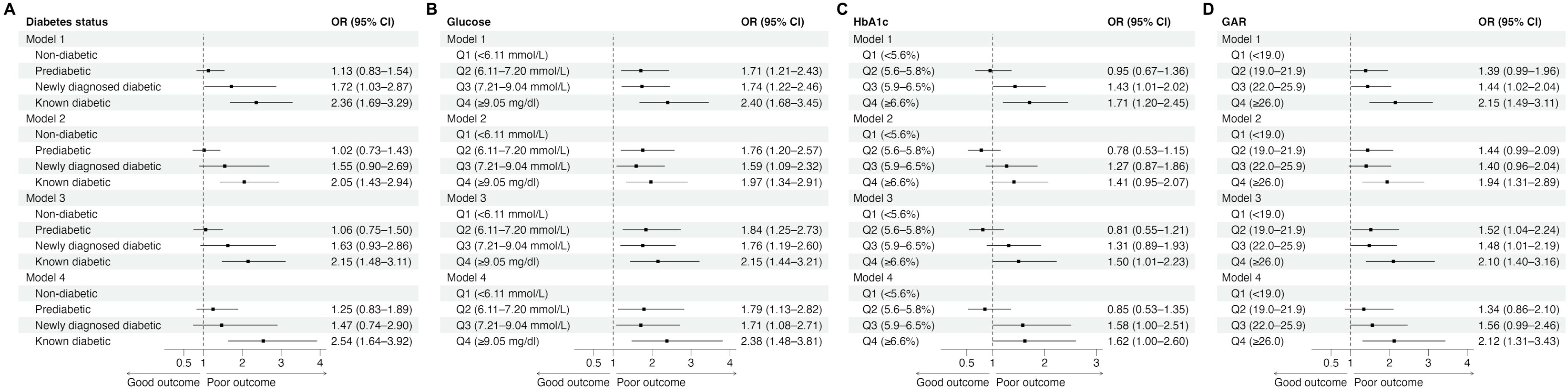

Supplementary Figure 1. The effects of diabetes status and glucose measures on 90-day functional outcomes in univariable (Model 1) and multivariable logistic regression analyses (Model 2: adjusted for age, sex, anterior circulation stroke, and NIHSS score; Model 3: adjusted for age, sex, anterior circulation stroke, NIHSS score, and reperfusion success; Model 4: adjusted for age, sex, anterior circulation stroke, NIHSS score, reperfusion success, and last known well to reperfusion time). CI, confidence interval; GAR, glucose-to-HbA1c ratio; HbA1c, glycated hemoglobin; OR, odds ratio.

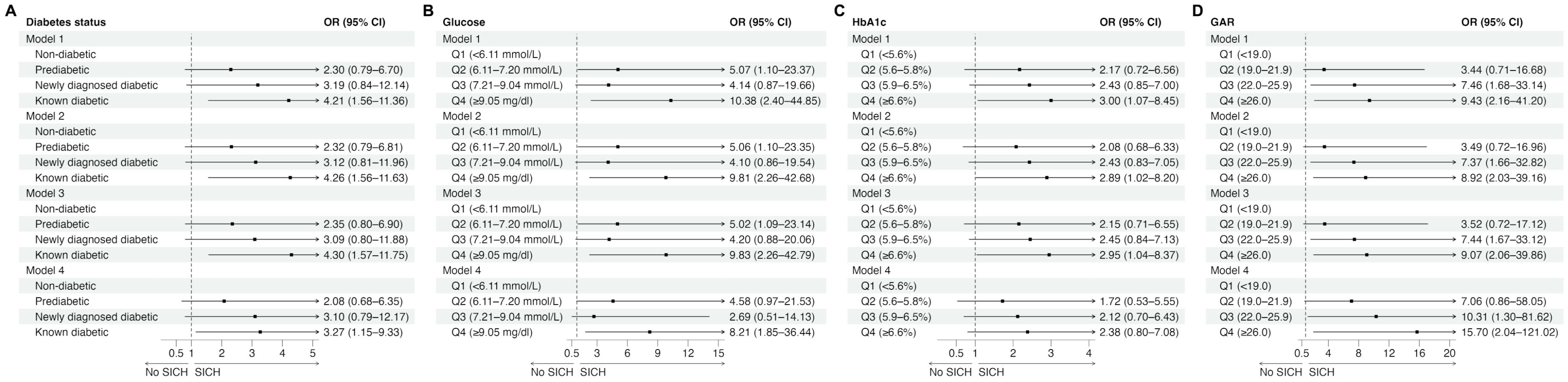

Supplementary Figure 2. The effects of diabetes status and glucose measures on symptomatic intracranial hemorrhage in univariable (Model 1) and multivariable logistic regression analyses (Model 2: adjusted for age, sex, anterior circulation stroke, and NIHSS score; Model 3: adjusted for age, sex, anterior circulation stroke, NIHSS score, and reperfusion success; Model 4: adjusted for age, sex, anterior circulation stroke, NIHSS score, reperfusion success, and last known well to reperfusion time). CI, confidence interval; GAR, glucose-to-HbA1c ratio; HbA1c, glycated hemoglobin; OR, odds ratio; SICH, symptomatic intracranial hemorrhage.

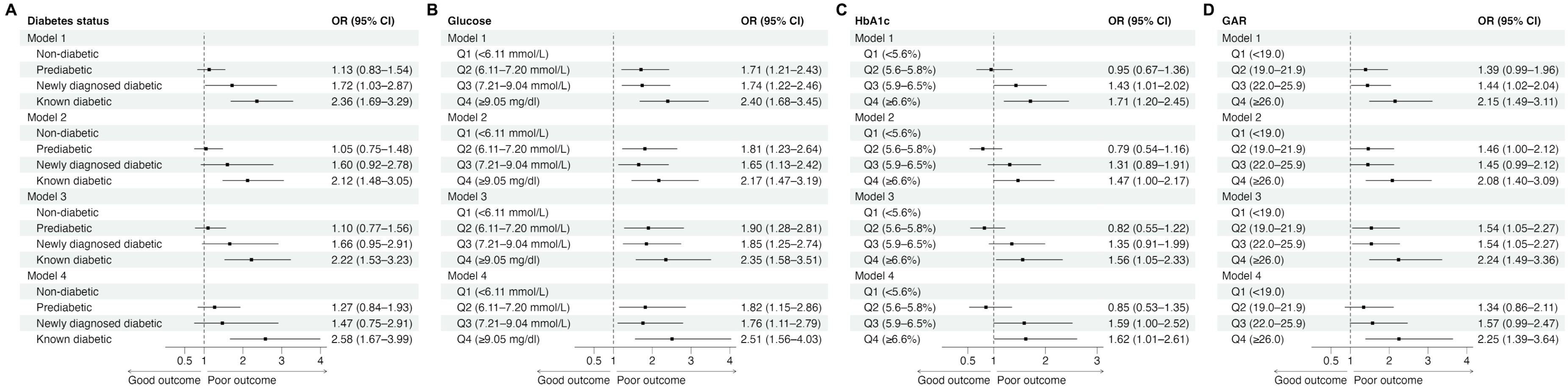

Supplementary Figure 3. The effects of diabetes status and glucose measures on 90-day functional outcomes in univariable (Model 1) and multivariable logistic regression analyses (Model 2: adjusted for age, sex, intravenous thrombolysis, and NIHSS score; Model 3: adjusted for age, sex, intravenous thrombolysis, NIHSS score, and reperfusion success; Model 4: adjusted for age, sex, intravenous thrombolysis, NIHSS score, reperfusion success, and last known well to reperfusion time). CI, confidence interval; GAR, glucose-to-HbA1c ratio; HbA1c, glycated hemoglobin; OR, odds ratio.

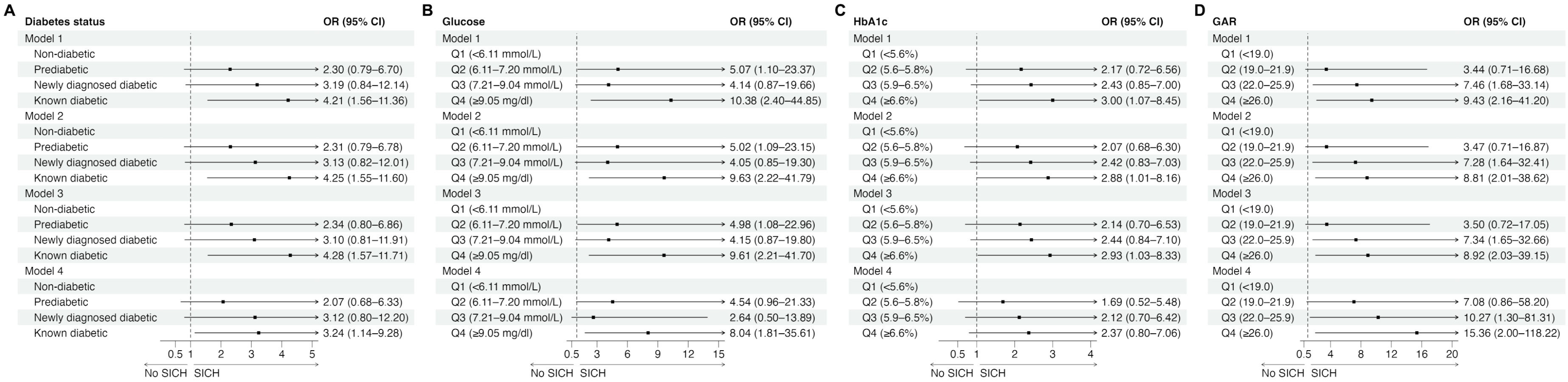

Supplementary Figure 4. The effects of diabetes status and glucose measures on symptomatic intracranial hemorrhage in univariable (Model 1) and multivariable logistic regression analyses (Model 2: adjusted for age, sex, intravenous thrombolysis, and NIHSS score; Model 3: adjusted for age, sex, intravenous thrombolysis, NIHSS score, and reperfusion success; Model 4: adjusted for age, sex, intravenous thrombolysis, NIHSS score, reperfusion success, and last known well to reperfusion time). CI, confidence interval; GAR, glucose-to-HbA1c ratio; HbA1c, glycated hemoglobin; OR, odds ratio; SICH, symptomatic intracranial hemorrhage.

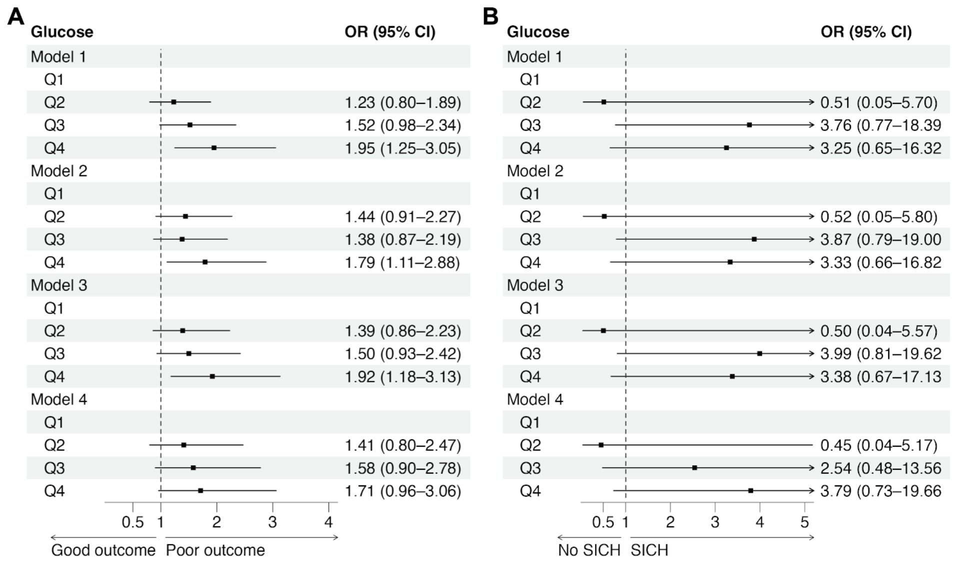

Supplementary Figure 5. The effects of admission glucose on 90-day functional outcomes (A) and symptomatic intracranial hemorrhage (B) in patients without diabetes in univariable (Model 1) and multivariable logistic regression analyses (Model 2: adjusted for age, sex, and NIHSS score; Model 3: adjusted for age, sex, NIHSS score, and reperfusion success; Model 4: adjusted for age, sex, NIHSS score, reperfusion success, and last known well to reperfusion time). CI, confidence interval; GAR, glucose-to-HbA1c ratio; HbA1c, glycated hemoglobin; OR, odds ratio; SICH, symptomatic intracranial hemorrhage.

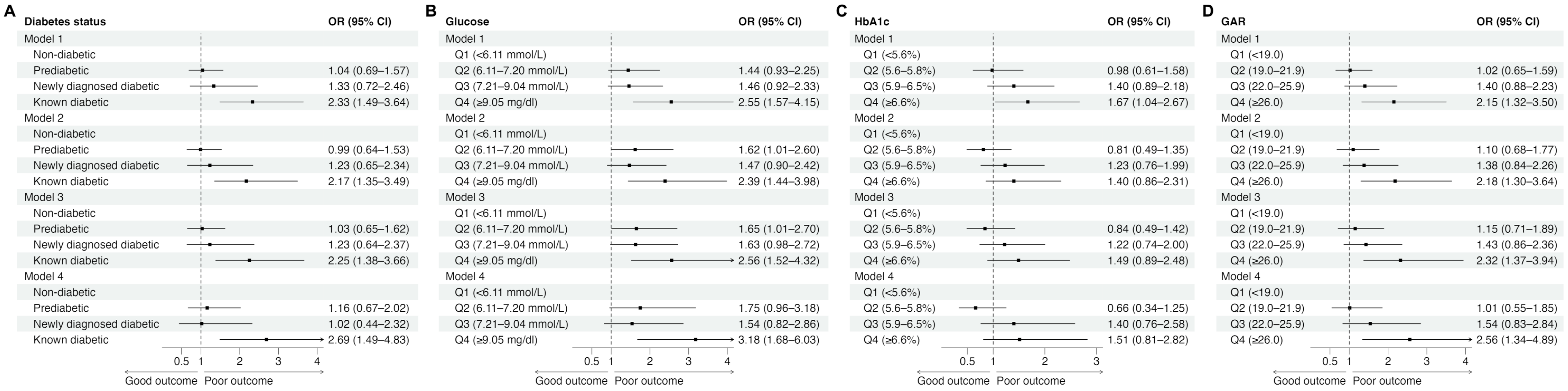

Supplementary Figure 6. The effects of diabetes status and glucose measures on 90-day functional outcomes in male patients in univariable (Model 1) and multivariable logistic regression analyses (Model 2: adjusted for age, and NIHSS score; Model 3: adjusted for age, NIHSS score, and reperfusion success; Model 4: adjusted for age, NIHSS score, reperfusion success, and last known well to reperfusion time). CI, confidence interval; GAR, glucose-to-HbA1c ratio; HbA1c, glycated hemoglobin; OR, odds ratio.

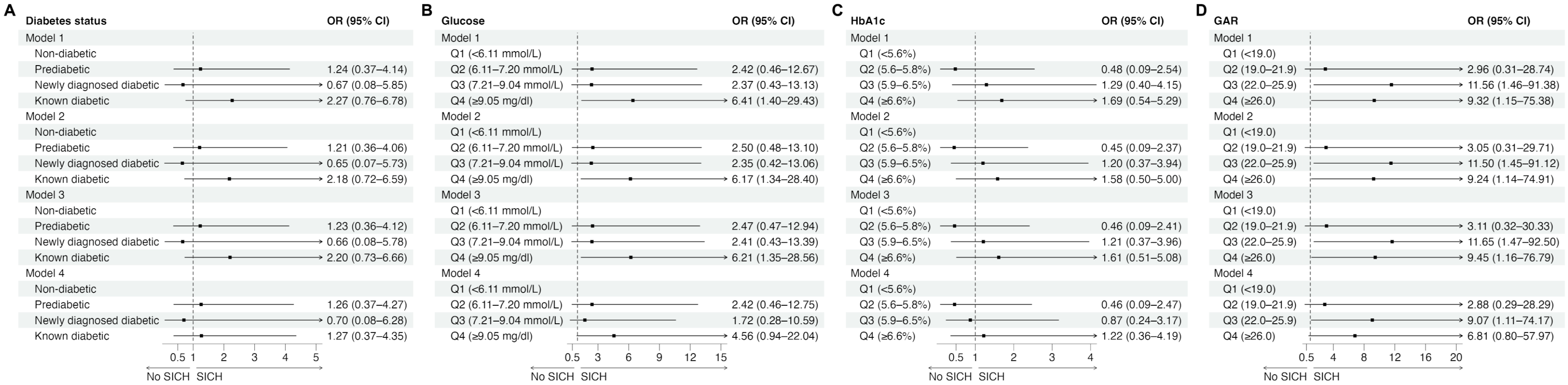

Supplementary Figure 7. The effects of diabetes status and glucose measures on symptomatic intracranial hemorrhage in male patients in univariable (Model 1) and multivariable logistic regression analyses (Model 2: adjusted for age, and NIHSS score; Model 3: adjusted for age, NIHSS score, and reperfusion success; Model 4: adjusted for age, NIHSS score, reperfusion success, and last known well to reperfusion time). CI, confidence interval; GAR, glucose-to-HbA1c ratio; HbA1c, glycated hemoglobin; OR, odds ratio; SICH, symptomatic intracranial hemorrhage.

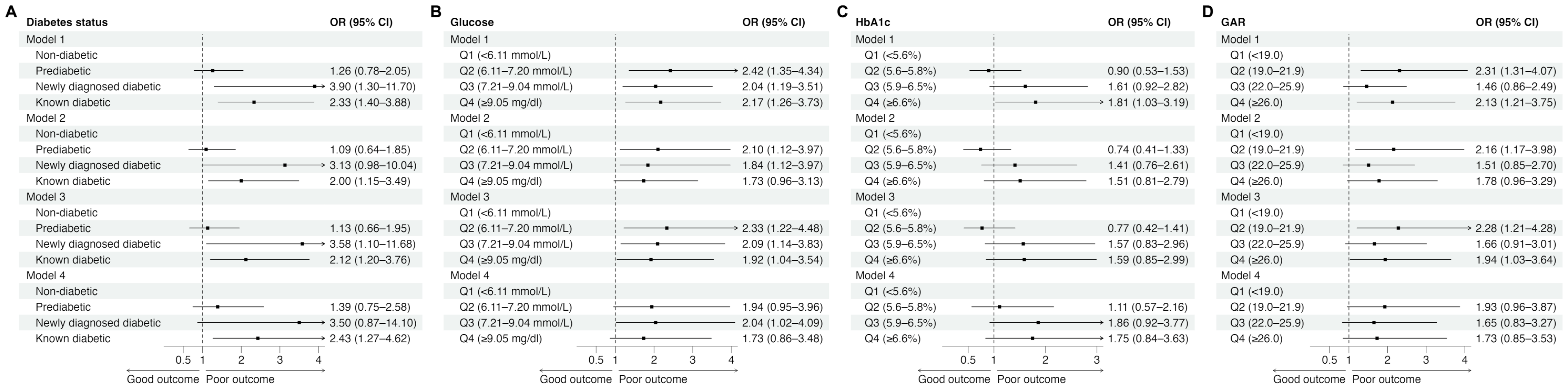

Supplementary Figure 8. The effects of diabetes status and glucose measures on 90-day functional outcomes in female patients in univariable (Model 1) and multivariable logistic regression analyses (Model 2: adjusted for age, and NIHSS score; Model 3: adjusted for age, NIHSS score, and reperfusion success; Model 4: adjusted for age, NIHSS score, reperfusion success, and last known well to reperfusion time). CI, confidence interval; GAR, glucose-to-HbA1c ratio; HbA1c, glycated hemoglobin; OR, odds ratio.

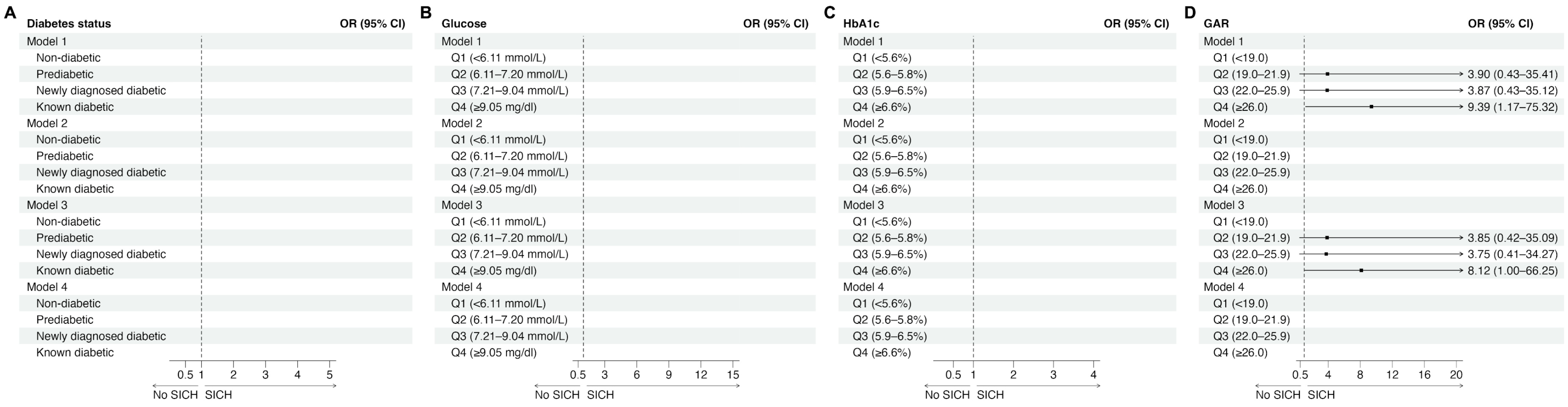

Supplementary Figure 9. The effects of diabetes status and glucose measures on symptomatic intracranial hemorrhage in female patients in univariable (Model 1) and multivariable logistic regression analyses (Model 2: adjusted for age, and NIHSS score; Model 3: adjusted for age, NIHSS score, and reperfusion success; Model 4: adjusted for age, NIHSS score, reperfusion success, and last known well to reperfusion time). CI, confidence interval; GAR, glucose-to-HbA1c ratio; HbA1c, glycated hemoglobin; OR, odds ratio; SICH, symptomatic intracranial hemorrhage.

Taiwan Registry of Endovascular Thrombectomy for Acute Ischemic Stroke (TREAT-AIS) investigators

National Taiwan University Hospital: Sung-Chun Tang (Principal Investigator), Jiann-Shing Jeng, Chung-Wen Lee, Chih-Hao Chen, Yen-Heng Lin, Shin-Joe Yeh, Bo-Ching Lee, Tai-Chun Chung

Taipei Veterans General Hospital: Chun-Jen Lin (Principal Investigator), I-Hui Lee, Nai-Fang Chi, Li-Chi Hsu, Chih-Ping Chung, Hung-Yu Liu, Chao-Bao Luo, Feng-Chi Chang, Chung-Jung Lin, Chia-Hung Wu, Kai-Wei Yu, Hsuen-En Hwang, Te-Ming Lin

Landseed International Hospital: Yu-Wei Chen (Principal Investigator), Chi-Jen Chen, Ching-Yi Wang, Yeh-Lin Kuo, Ping-Sheng Lu, Yen-Tung Chao, Yi-Hsin Su, Pei-Ju Lin, Yi-Chun Chen, Li-Ling Fan, Ju-Fang Yang

Chi Mei Medical Center: Kuan-Hung Lin (Principal Investigator), Chien-Jen Lin, Sheng-Hsiang Yang, Chun- Ming Yang, Huey-Juan Lin, Poh- Shiow Yeh, Chia- Yu Chang, Tian- Junn Cheng, Wei-Jia Lee, Ching-Chung Ko, Yu-Kun Tsui, Yun-Ju Shih, Te-Chang Wu, Meng-Tsang Hsieh

National Cheng Kung University Hospital: Pi-Shan Sung (Principal Investigator), Yu-Ming Chang Chun-Min Wang, Chih-Yuan Huang, Chih-Hung Chen

E Da Hospital: Te-Yuan Chen (Principal Investigator), Chang-Hsien Ou, Wan-Ching Lin, Li-Ching Chen, Bi-Shin Ann

Far Eastern Memorial Hospital: Chih-Wei Tang (Principal Investigator), Yen-Jun Lai, Lih-Wen Huang, Ya-Ling Kuo, Szu-Hsiang Peng, Yi-Chun Pai Lin

En Chu Kong Hospital: Hai-Jui Chu (Principal Investigator), Cheng-Huai Lin, Yu Sun, Chien-Jung Lu, Chun-Yu Lee, Chang-Hsiu Liu

National Taiwan University Hospital Hsin Chu Branch: Kun-Chang Tsai (Principal Investigator), Kuo-Wei Chen, Li-Kai Tsai, Yen-Chung Hsiue, Ya-Wen Cheng, Chuan-Hsiu Fu, Wen-Yu Chen

Mackay Memorial Hospital: Chao-Liang Chou (Principal Investigator), Helen L. Po, Ya-Ju Lin, Yung-Pin Hwang, Shu-Fan Kuo, Chun-Chao Huang, Zong-Yi Jhou, Hui-Fen Yu, Hsiao-Chu Lin

Chang Bing Show Chwan Memorial Hospital: Cheng-Yu Wei (Principal Investigator), Chih-Lin Chen, Pei-han Wu, Yi-Ching Tsai

Tri Service General Hospital, National Defense Medical Center: Shang-Yih Yen (Principal Investigator), Jiunn-tay Lee, Chung-Hsing Chou, Chien-An Ko

Taichung Veterans General Hospital: Po-Lin Chen (Principal Investigator), Yuang-Seng Tsuei, Wen-Hsien Chen, Nien-Chen Liao, Yeng-Fung Liaw

Shin Kong WHS Memorial Hospital: Hsu-Ling Yeh (Principal Investigator), Li-Ming Lien, Chen-Yu Hsiao, Kuan-Yu Lin, Tsui-Hua Yang

Taipei Medical University Shuang-Ho Hospital: Lung Chan (Principal Investigator), Jia-Hung Chen, Shun-Fan Yu, I-Chang Su, Yueh-Hsun Lu

Ditmanson Medication Foundation Chia-Yi Christian Hospital: Sheng-Feng Sung (Principal Investigator), Tzu-Hsien Yang, Chu-Hsien Lin, Yung-Chu Hsu, Yu-Hsiang Su, Ling-Chien Hung, Mao-Hsun Lin, Chien-Yu Su

Fu Jen Catholic University Hospital: Hon-Man Liu (Principal Investigator), Yung-Chuan Huang, Chih-Cheng Wan

Kaohsiung Veterans General Hospital: Ching-Huang Lin (Principal Investigator), Cheng-Chang Yen, Ching-Sen Shih

Chiayi Chang Gung Memorial Hospital: Meng Lee (Principal Investigator), Yuan-Hsiung Tsai, Yen-Chu Huang, Wei-Tse Hung, Jiann-Der Lee
